# Current Achievements and Applications of Transcriptomics in Personalized Cancer Medicine

**DOI:** 10.3390/ijms22031422

**Published:** 2021-01-31

**Authors:** Stanislaw Supplitt, Pawel Karpinski, Maria Sasiadek, Izabela Laczmanska

**Affiliations:** 1 Department of Genetics, Wroclaw Medical University, Marcinkowskiego 1, 50-368 Wroclaw, Poland; polemiraza@poczta.fm (P.K.); maria.sasiadek@umed.wroc.pl (M.S.); izabela.laczmanska@umed.wroc.pl (I.L.); 2 Laboratory of Genomics and Bioinformatics, Hirszfeld Institute of Immunology and Experimental Therapy, Polish Academy of Sciences, Weigla 12, 53-114 Wroclaw, Poland

**Keywords:** transcriptomics, RNA, cancer, oncogenesis, personalized medicine

## Abstract

Over the last decades, transcriptome profiling emerged as one of the most powerful approaches in oncology, providing prognostic and predictive utility for cancer management. The development of novel technologies, such as revolutionary next-generation sequencing, enables the identification of cancer biomarkers, gene signatures, and their aberrant expression affecting oncogenesis, as well as the discovery of molecular targets for anticancer therapies. Transcriptomics contribute to a change in the holistic understanding of cancer, from histopathological and organic to molecular classifications, opening a more personalized perspective for tumor diagnostics and therapy. The further advancement on transcriptome profiling may allow standardization and cost reduction of its analysis, which will be the next step for transcriptomics to become a canon of contemporary cancer medicine.

## 1. Introduction

Cancers are a highly heterogeneous group of diseases whose sensitivity to the therapy varies considerably among patients [1]. The commonly accepted clinical principle attributes the histological type of tumor and its stage, according to the tumor-node-metastasis (TNM) system, as the causes of differences in the treatment response [2,3]. However, the understanding of cancer biology has changed significantly over the last years due to the prominent progress in genomics. Since the completion of the Human Genome Project and development of next generation sequencing (NGS) techniques, the structure and functions of the genome, as well as mechanisms regulating genes’ expression have been extensively explored in the context of oncology [4,5]. The constantly widening knowledge on genomic background of malignant transformation brings a new insight into the molecular justification of pathogenesis of particular tumors and their targeted treatment [6,7].

The branch of molecular genetics with remarkably fast-paced development over the recent years is transcriptomics [4,8]. A transcriptome is a set of all the RNA molecules transcribed from the genome in a given cell, at a particular developmental stage and under certain physiological or pathological conditions [9,10]. It includes protein-coding RNAs (pcRNAs), typically referred to as messenger RNAs (mRNAs), and non-coding RNAs (ncRNAs), from which every particular molecule presents different spectrum of functions in the cell and reacts variously to environmental stimuli [11,12,13,14]. The transcriptome profile can be regarded as a snapshot of the temporary cell state and thus, its analysis provides not only information on gene function, but also reveals details on the genome plasticity, gene expression regulation, and modifications of individual transcripts [15]. Therefore, transcriptome analysis is considered as a useful approach for investigation of constantly changing cancer cells at a molecular level. Obtained conclusions contribute directly to the intensive development of precision oncology [16,17,18].

The article summarizes the current achievements of transcriptomics in oncology with an emphasis on its potential in guiding diagnostics and anticancer therapies.

## 2. Omics: Different Levels of Gene Expression

**Figure 1:**
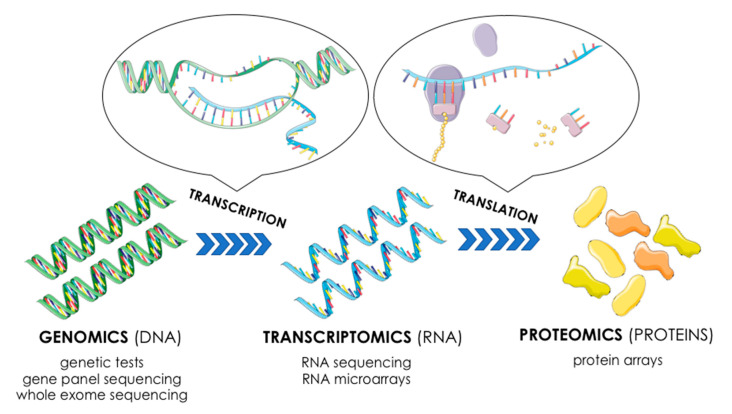
Omics—different approaches to gene expression (based on [22]). This figure was created using images from Servier Medical Art Commons Attribution 3.0 Unported License. (http://smart.servier.com). Servier Medical Art by Servier is licensed under a Creative Commons Attribution 3.0 Unported License.

The knowledge on the genetic principles of neoplastic transformation enables its application both for molecular and clinical purposes [19]. The development of cancer is always associated with genetic and epigenetic changes accumulated within the cell, through which it acquires aberrant biological features specific for cancer cells (e.g., loss of apoptosis, insensitivity to regulatory factors, uncontrolled growth and cell division) [20,21]. The genetic alterations and global changes in gene expression can be considered at three distinct levels, i.e., genomic, transcriptomic, and proteomic (Figure 1), where every level presents respective unique advantages and limitations. The analysis of complex interactions among the above-mentioned molecular levels forms the basis for understanding of personalized oncology [22].

The genomic level can be regarded as the farthest from the cellular phenotype. Since the human genome was sequenced, the major progress has been made in research of mutations driving cancer cells [23]. Specific mutations responsible for malignant transformation and hereditary cancer syndromes were identified [24]. Despite the advantages of DNA sequencing, the mutation assays present strong limitations in personalized medicine [25]. First of all, only a small percentage of human genome is expressed to the protein level. Secondly, gene expression is a very complicated and multistage process, controlled by numerous regulating mechanisms, such as DNA methylation [26], DNA-binding proteins [27], or small interfering RNA (siRNA) [28]. Moreover, molecular studies, as well as karyotyping and genomic hybridization revealed cancer cell heterogeneity. It was discovered that cells within the same tumor may vary so significantly in terms of DNA sequence to the point of defining different subpopulations, implicating clinical heterogeneity [29,30]. Furthermore, plasticity of cancer genomes manifesting itself in cell-to-cell DNA sequence variability (observed during tumor development and induced by treatment), precludes the achievement of large-scale clinical benefits [31]. In conclusion, the analysis of cancer genome may provide valuable information on the DNA sequence and its structure, but could be inadequate to describe the actual phenotype of the cell [22]. Therefore other approaches are needed to find proper molecular diagnostic targets and to address specific therapy for cancer patients.

The proteomic approach, which is obviously closer to the molecular mechanisms determining cell’s phenotype than genomics [32], focuses on quantitative protein measurements to characterize biological processes and deciphers the protein-dependent mechanisms of gene expression regulation in a living cell [33,34]. It is confirmed that proteins are the key factors in all cellular processes, while mutations alter their expressions and/or activities in various ways [35,36]. The analysis of the cancer proteome enables to obtain a landscape of post-translational modifications, interactions between cellular mechanisms and their locations [37]. Among techniques of protein recognition, microarrays are the most frequently employed. This method uses monoclonal antibodies or other binders for identification of an individual protein. Protein microarrays have gained extensive applications in molecular diagnostics, particularly in cancer biomarker discovery [33]. Nevertheless, technical difficulties related to the method, such as different physical and chemical properties of proteins, as well as the need of usage of highly specific antibody panels, are the significant obstacles for proteomic studies in a wide range [38].

The pivotal link between cellular phenotype and genetic aspects of tumor biology is transcriptome. It contains all information encoded in RNA transcribed from DNA. In contrast to the genome which is relatively stable, transcriptome reacts actively to the physiological or pathological conditions. Adjustments in the transcriptome reflect the different cell states, developmental stages, and regulatory mechanisms [15]. Nowadays, transcriptomics have come to the forefront of international scientific attention due to the rapid development of RNA-sequencing (RNA-Seq) methods.

## 3. Transcriptome’s Components and Methods of Its Analysis

Studies show that about 93% of the human DNA is transcribed into RNA, however only 2% into pcRNA [4]. These molecules, in a mature form, specify the sequence of amino acids in the protein and participate in translation. The remaining part of the human transcriptome consists of ncRNA and, out of all discovered until now, include: ribosomal RNA (rRNA), transfer RNA (tRNA), micro RNA (miRNA), small interfering RNA (siRNA), PIWI-interacting RNA (piRNA), small nuclear RNA (snRNA), small nucleoar RNA (snoRNA), extra-cellular RNA (exRNA), guide RNA (gRNA), small Cajal body-specific RNA (scaRNA), circular RNA (circRNA), long non-coding RNA (lncRNA), such as X-inactive specific transcript (XIST) and HOX Transcript Antisense RNA (HOTAIR) [39]. Their cellular functions significantly differ from each other. Some ncRNAs present catalytic roles, e.g., involved in production of tRNA and rRNA; whereas other participate in controlling mRNA, e.g., miRNA, snRNA, snoRNA [40].

All the above-mentioned ncRNA types potentially contribute to oncogenesis. Moreover, previously unknown types of RNAs are being continuously discovered—mostly due to modern technologies. Meticulous analysis of RNAs’ functions and the development of novel techniques may lead to the improvement of transcriptome cognition.

### 3.1. History of Gene Expression Measurement Methods

Several technologies have been developed to define the role of all RNAs forming transcriptome in human diseases. The first approach of studying human transcriptome ascribes to quantification of mRNA with Northern blotting [41,42]. This time-consuming method uses gene-specific DNA probes which are hybridized to the RNA and is capable of analyzing a maximum a few gene transcripts at the same time [43].

Subsequently newer methods based on complementary probe hybridization were introduced to transcriptome analysis [8]. Today, three main directions can be distinguished in cancer transcriptomics: microarrays, large-scale quantitative reverse-transcription-polymerase chain reaction (qRT-PCR) (both using probe hybridization), and the newest, next-generation sequencing (NGS) technology, represented by RNA-Seq [8,44].

#### 3.1.1. Microarrays

**Figure 2:**
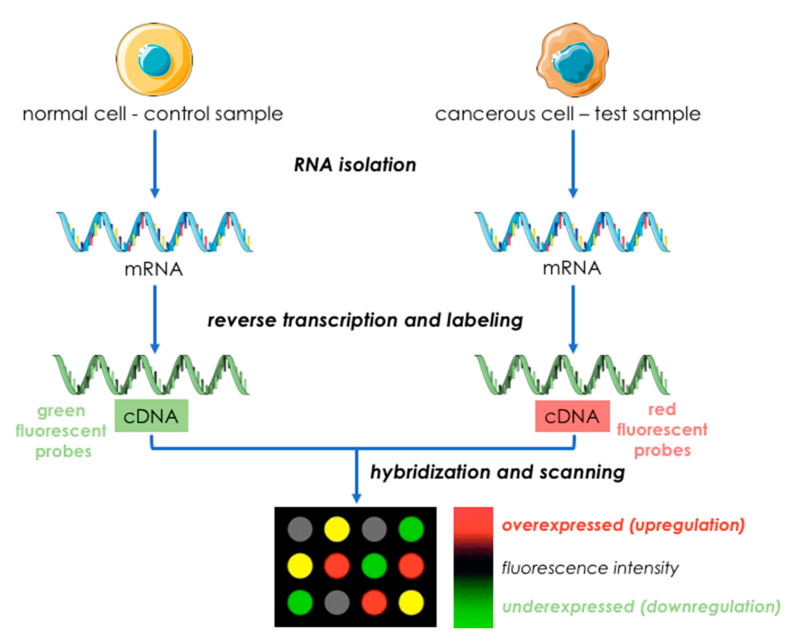
An overview of DNA microarray technology (based on [47]). This figure was created using images from Servier Medical Art Commons Attribution 3.0 Unported License. (http://smart.servier.com). Servier Medical Art by Servier is licensed under a Creative Commons Attribution 3.0 Unported License.

Microarray technology gained wide approval since its discovery in mid-1990s and this method quickly became the most frequently used for transcriptome profiling [45,46]. A microarray is a collection of microchips of microbeads containing DNA probes (nucleotide oligomers) corresponding to known sequences. RNA is isolated from the control and the target samples, undergo reverse transcription and labeling and then cDNA is hybridized to a microarray. The abundance of hybridization is quantified by fluorescently labelled probes and the results are adapted to measure the expression levels [47] (Figure 2). Microarrays found a wide usage also in the research of tumor mRNA, particularly enabled the identification of differentially expressed genes involved in key molecular pathways and discovery of numerous cancer biomarkers [48,49]. Although the measurement of mRNA down- or upregulation with microarrays is a powerful tool for analyzing gene expression and molecular behavior of transcriptome, the results may vary significantly depending on microarray platforms, type of laser scanners, laboratories procedures and analytical methods [50]. Therefore, analysis of mRNA expression with microarrays should be considered as a preliminary step, and verified by RT-PCR or immunohistochemical analysis [51].

#### 3.1.2. Large-Scale Real-Time Reverse Transcription PCR

Another technique that was involved in transcriptome profiling is large-scale real-time (quantitative) reverse transcription PCR (RT-PCR). The method was first successfully applied for verification of microarray RNA analysis, and thereafter it has been extended to become a self-sufficient technique as it is fast, reliable and enables the accurate and simultaneous profiling of many genes expression [4,52]. It is noteworthy that RT-PCR found broad applications in diagnostics and treatment monitoring in oncology [53]. It has been used in research of prognostic and predictive tumor biomarkers, e.g., in breast cancer [54], melanoma [55,56], or hepatocellular carcinoma [57,58], as well as for other purposes, such as micrometastasis determination [59] or detection of circulating tumor cells [60,61]. In this method complementary DNA (cDNA) is generated from RNA template in reverse transcription and specific DNA targets are amplified using polymerase chain reaction (PCR), employing large set of primer pairs. Quantitative PCR enables to evaluate the increase in copies of investigated sequences in real time. It is possible due to labelling of primers, oligonucleotides, or amplification products with fluorophores. A positive reaction is detected by the accumulation of a fluorescent signal.

#### 3.1.3. RNA-Sequencing (RNA-Seq)

Above-mentioned hybridization-based methods are limited by the need of prior knowledge on the sequence of the probed nucleic acid [62]. Therefore, transcriptomics evolved to sequencing-based techniques including consecutively: Sanger sequencing of expressed sequence tags (ESTs) and NGS.

Sanger sequencing is a method of controlled termination of DNA synthesis [63]. A sample matrix is usually single-stranded DNA amplified in PCR [64]. The sequencing process requires the sample matrix, DNA polymerase, one primer, four deoxynucleotidetriphosphates (dNTPs) and four fluorescent labelled dideoxynucleotidetriphosphate (ddNTPs). DNA polymerase is applied for synthetizing nucleic acid strand using either dNTPs, which enables continuation of synthesis, or ddNTPs, which terminates further synthesis in a specific locus [65]. For the purpose of RNA studies, ESTs—short sub-sequences of cDNA complementary to a part of mRNA—are used with Sanger sequencing [8]. Before NGS technology was introduced, tag-based sequencing had been commonly used in the research of tumor biomarkers [66,67,68]. Nevertheless, it presents significant limitations: it can sequence short pieces of DNA—up 1000 base pairs, the primer’s template must be defined and there has to be large amount of copies of the gene in the starting mixture for sequencing [69].

#### 3.1.4. Next Generation Sequencing (NGS)

The new era of transcriptome profiling is set by the NGS. It provides abundant possibilities to perform simultaneous investigations of thousands of genes, analyses of complicated molecular mechanisms participating in oncogenesis and thus, offers a great contribution to the precision medicine. NGS technology, first introduced in 2004, may be applied in various areas: whole-genome and exome sequencing, RNA-Seq, which is referred today as a “gold standard” of transcriptome analysis, chromatin immunoprecipitation followed by sequencing (ChIP-Seq) for analysis of interactions between DNA and proteins and copy number variation-sequencing (CNV-Seq) to analyze DNA copy number variation [70]. RNA-Seq enables high-throughput gene expression analysis with remarkable precision. NGS generates data about splicing variants, allelic expression and RNA editing [71]. It enables the analysis of epigenetic modifications and elements playing role in mechanisms, such as ncRNAs [72]. This technology has significantly increased the efficiency, sensitivity, accuracy and transcript coverage in obtained results in comparison to microarrays [73].

Several NGS platforms are based on the pyrosequencing principle, where binding of nucleotides during chain synthesis is monitored by luminescence [74]. After the incorporation of a specific dNTP, a lightning signal is generated when pyrophosphate (PPi) is converted to ATP by ATP sulfurylase in the presence of adenosine 5′ phosphosulfate (APS). Two sequencing platforms directly utilize pyrosequencing principle: 454 Sequencing (454 Life Sciences; now Roche Holding AG, Basel, Switzerland) and PyroMark ID system (Qiagen, Hilden, Germany) [75,76]. Other massive parallel sequencing instruments include: Illumina-Solexa Genome Analyzer family launched in 2006, Ion Torrent, Thermo Fisher and Bionano Technologies [77]. Considering main differences between analyzers, Roche display higher length reads in comparison to Illumina, but produce more errors within homopolymer regions [78,79].

RNA-seq has been widely used for the research on gene expression in various malignancies and has been found as an effective tool in exploration of carcinogenesis, identification of tumor biomarkers and development of new therapeutic strategies. The following parts of the article provide a clearer image of the achievements of transcriptomics over the recent years.

## 4. Applications of Transcriptome Analysis in Oncology

The transcriptome-based studies opened a new chapter in molecular understanding of cancer. Analysis of gene expression allows researchers to explore molecular basis of cancer in a multiway manner and provides information of tumor progression. Technologies involved in transcriptomics have expanded our knowledge on carcinogenesis, tumor microenvironment (TME), and immuno-oncology, as well as enabled the discovery of new, previously unknown cancer biomarkers.

### 4.1. Applications in Clinical Classifications of Cancer

Identification of the molecular portrait of the tumor is valuable in cancer classification and elucidation of biochemical pathways, which allows further selection of selective therapeutics and prediction of cancer drug sensitivity [80]. Analysis of the transcriptome has led to a significant improvement in clinical classification of many tumors [81,82].

So far, numerous studies have been carried out to profile gene expression and thus, to establish molecular classification and identification of therapeutic/diagnostic targets in breast cancer [83,84,85,86]. One of the first successful attempts of gene expression-based characterization was made by Perou et al. (2000). In 65 samples of breast cancer obtained from 42 patients, unique patterns of gene expression were observed for every individual [87]. Nevertheless, four different molecular clusters of breast cancer, presenting similar expression signatures, were captured: ER+/luminal-like, basal-like, Erb-B2+ (Her-2/neu) and normal-like breast tumors. The clusters distinguished in this study correspond closely to immunohistochemistry markers of breast tumors: estrogen receptors, Her-2 and Ki-67 [88], and thus, should be treated as distinct diseases [89,90]. Moreover, similar conclusions were drawn in the study of Sørlie et al. [91], where luminal type of breast cancer was divided into two subtypes: luminal A (with high estrogen receptor expression and low expression of proliferative markers, e.g., Ki67) and luminal B (presenting lower estrogen receptor expression and high level of expression of proliferation-related genes) [92,93]. The above-mentioned studies were carried out by using microarray technology, which laid the foundations of current clinically used molecular classification of breast cancer [94].

Analogous efforts were made in classification of colorectal cancer (CRC). In the research conducted by Guinney et al., four consensus molecular subtypes of CRC were distinguished: CMS1 (microsatellite instability immune-hypermutated, characterized by strong immune activation, microsatellite unstable), CMS2 (canonical—marked by WNT and MYC signaling pathways activation), CMS3 (metabolic—epithelial with evident metabolic dysregulation), and CMS4 (mesenchymal—with growth factor–β activation, stromal invasion and angiogenesis) [95]. Recently, many clinical trials demonstrated the usability of this classification, confirming different sensitivities to the treatment, recurrence parameters and tendencies to metastasis of individual CMS subtypes [96,97,98,99,100]. Such an approach aids in the understanding of CRC etiology for the purposes of precision oncology. However, further population-based trials must be performed to prove the advantages of introducing CMS classification into clinical oncology [84,101].

Another study uncovered differences in origin of ovarian cancer. It is pathologically divided into subtypes: epithelial (EOC), sex-cord stromal and germ cell, where EOC is subdivided into: high-grade serous, low-grade serous, mucinous, endometrioid and clear cell carcinoma [102]. According to the classic view on high-grade serous ovarian cancer (HGSOC), it originates from epithelial cells of ovarian surface. However, recent studies show that tumor may develop from fallopian tube [103,104]. The molecular profiling of HGSOC has revealed overexpression of PAX8, which is typical for cells within the distal fallopian tube [105,106]. The determination of PAX8 overexpression by using RNA-Seq may accelerate diagnostics.

Other attempts of classifying cancers based on their gene expression signatures have been made (among others) in uveal melanoma [107], small round-blue cells tumors (neuroblastoma, rhabdomyosarcoma, non-Hodgkin lymphoma, Ewing tumor) [108], large cell lung cancer [109], and head and neck squamous cell carcinoma [110] (Table 1).

### 4.2. Identification of Early Detection Cancer Biomarkers

There is an urgent need to find new early diagnosis and treatment monitoring biomarkers for most of tumors. For instance, the usefulness and clinical reliability of carbohydrate antigen 125 (CA125), which is the most commonly utilized serum biomarker of ovarian cancer remain controversial [114]. The specificity of CA125 ranges between 35% and 91% [115]. CA125 level is raised only in approximately 50% of early onset ovarian cancer cases and up to 90% at advanced stage [116]. The study of Mosig et al., performed with the RNA-Seq-based transcriptome analysis of 22 samples from ovarian cancer patients, showed significant overexpression of insulin-like growth factor binding protein-4 (*IGFBP-4*). The IGFBP-4 transcript level was elevated in initial and advanced disease phase as well as in relapse samples, regardless of CA125 levels [117].

Another example of utilization of gene expression profiling in the selection of cancer biomarkers can be found in gastrointestinal tract tumors. Despite the development of multidisciplinary treatment of esophageal cancer, the clinical outcomes often remain insufficient due to late diagnosis of the disease [118]. Hence, the discovery of relevant biomarkers enabling early screening is of high importance. Currently, the most commonly used biomarkers in esophageal squamous cell carcinoma (ESCC) diagnosis are: cytokeratin 19 fragment antigen 21–1 (Cyfra21–1), carbohydrate antigen 19–9 (CA19–9), carbohydrate antigen 72–4 (CA72–4), carcinoembryonic antigen (CEA), and squamous cell carcinoma antigen (SCC-Ag) [119]. The above-mentioned proteins are characterized by high specificity for ESCC, but low sensitivity ranging between 10% and 40% [120]. Recent approaches combine mRNA sequencing or proteome sequencing together with computational analysis, have identified highly expressed genes or highly secreted proteins, which may serve as early detection biomarkers. Bioinformatics enables parallel analysis of thousands of variables in the genome-wide scale, assess their significance, which facilitates selection of relevant variables and allows for biologically meaningful interpretations [121]. However, chemistry, sequencing, and data preprocessing differ significantly between proteome sequencing and mRNA sequencing, the downstream steps are similar [122]. Statistical comparisons of normal tissue, benign neoplasia and tumor tissue by data mining methods represent a common way of searching and prioritization of biomarkers [123]. Regarding statistical analyses used to compare various stages of neoplasia, there are a huge variety of data mining methods including relatively simple feature (gene) selection algorithms (e.g., t-test, principal component analysis (PCA), nucleoside triphosphate (NTP) classifier and least absolute shrinkage and selection operator (LASSO)) or more mathematically sophisticated approaches (e.g., support vector machine (SVM)-based, Bayesian network-based, neural network-based, or ensemble-based) [124]. It has to be emphasized that difference in terms of performance (specificity, sensitivity, accuracy) is comparable between these two classes of mining methods (simple and sophisticated). Therefore, in current biomarker research simple solutions such as NTP classifier became popular [125,126]. Moreover, sets of selected markers offer much better performance than single marker, therefore, currently in various studies predictive power have benefited from such multi-marker approach [127]. For example, in the study of Xing et al., three serum markers were evaluated as potentially clinically usable: CHI3L1, MMP13, and SPP1, from more than 4000 differentially expressed genes in the ESCC transcriptome database. The diagnostic panel using combination of CHI3L1, MMP13 and SPP1 identified approximately 90% of cases with early-onset ESCC, where the panel’s detectability in non-cancerous tissues ranged between 10% and 15% [128].

Moreover, various RNA molecules may serve as independent cancer biomarkers [129]. Over the recent years, many RNAs were found to be useful in precision oncology (Table 2). For instance, the expression profile of snoRNAs [130] and 4-miRNA expression signature [131] can be used in early diagnosis of non-small-cell lung carcinoma (NSCLC). In addition, miR-106b, miR-20a, and miR-221 were confirmed as early detection biomarkers for gastric cancer [132,133]. The piRNAs, which mediate transcriptional and post-transcriptional gene silencing machineries, may serve as biomarkers for early diagnosis, treatment and prognosis of: renal cancer [134,135], hepatocellular carcinoma [136], glioblastoma [137] and gastric cancer [138]. Moreover, lncRNAs, such as XIST, present as potential candidates for the detection of early gastric cancer [139]. A growing number of studies have indicated the possibility of applications of ncRNAs in CRC as well [140,141].

The cancer cells for molecular analyses may be obtained directly from cancer tissue, from peripheral blood [159] and (depending on the tumor primary locus) from other specimens, e.g., sputum [160,161]. In recent years, an emerging field of future cancer diagnostics is liquid biopsy—unique sampling of neoplastic material from the blood. Two main directions can be distinguished for studies on liquid biopsy: analysis of circulating tumor cells (CTCs) and analysis of circulating nucleic acids. The first approach is limited by the uncommonness of cancer cells in the bloodstream (around one CTC per billion blood cells) [162], which significantly reduce the clinical application of this method. Alternatively, analysis of nucleic components of tumor cells—cell free DNA (cfDNA) and circulating RNA (circRNA, including miRNA)—is gaining an increasing interest, demonstrated in numerous studies and clinical trials [159,163,164,165]. Rapid advancement of novel technologies, enables to detect cancerous DNA/RNA with high accuracy for diagnosis, prognosis and therapeutic monitoring [166]. RNA monitoring, in comparison to DNA-based biopsy, presents two main advantages: it is a better tool for identification of gene fusions and functional disease profiling, through detection of ncRNA of biological importance and characterization of alternative RNA splicing products [167,168]. Consequently, a number of recent studies involving RNA obtained from liquid biopsy is steadily increasing (mostly for miRNA markers). Results of large, carefully conducted validation studies (sample sizes above 500) showed promising opportunities of applying miRNA signatures in clinics in the near future. For example, miR-Test published in 2015 is based on signature of 13 serum miRNAs for early and sensitive detection of lung cancer. MiR-Test displayed the sensitivity of 77.8% and the specificity of 74.8% to detect lung tumors [169]. There are also several urine-based tests for detection of prostate cancer including SelectMDx (based on gene expression signature) and Progensa PCA3 (based on lncRNA expression signature) [170,171]. Both tests revealed their potential in elimination of unnecessary prostate biopsies [172].

### 4.3. Creation of Cancer Prognostic and Predictive Panels

Various assays based on gene expression analysis have been developed to guide personalized therapeutic pathway in oncology [22]. Multigene tests provide detailed insight into cancer biology with simultaneous information on expression of relevant, prognostic fundamental genes. Some assays are already used in diagnosis, as prognostic panels and for the prediction of treatment effects (mostly adjuvant or neoadjuvant systemic therapy) [173].

Among most commonly used gene expression tests are MammaPrint^®^ and Oncotype DX^®^ assessing the risk of relapse and metastasis in breast cancer. MammaPrint^®^ is 70-gene panel, tested in large randomized controlled trial (MINDACT, ClinicalTrials.gov identifier: NCT00433589), where its ability to differentiate low-risk and high-risk patients with breast cancer was confirmed [174,175]. Oncotype DX^®^ is 21-gene qRT-PCR test assessing the risk of breast cancer recurrence, with particular emphasis on early-stage, ER-positive (estrogen receptor), HER2-negative and axillary node-negative tumors [176]. The advantages of Oncotype DX^®^ panel were presented widely in trial TAILORx (ClinicalTrials.gov identifier: NCT00310180) [177]. Other transcriptomic signatures of breast cancer with clinical usage are: Prosigna^®^-Assay (PAM50—50-gene panel) [178,179], Endopredict^®^ (12-gene assay) [180,181].

The similar assays are being tested in prognosis of colon cancer. The twin test of Oncotype DX^®^ used in breast cancer – 12-gene Oncotype DX^®^ Colon Cancer Assay identifies the tumors with high risk of recurrence and helps to determine the necessity of implementation of adjuvant systemic therapy [182,183,184]. In the study of Aziz et. al, the 19-gene signature was evaluated as a predictive classifier in colorectal cancer using microarray profiling [185]. It was revealed that the patients assigned into the low-risk group could avoid or receive lower concentrations of adjuvant chemotherapy.

Afirma^®^ gene expression classifier is a microarray assay used for thyroid cancer diagnosis. It is validated as pre-operative test for differentiating invasive cancer from benign nodules (such as follicular adenoma) in order to avoid unnecessary surgeries [186]. The cytological tests used routinely for attaining that purpose are characterized by frequent false results, therefore genetic testing is valuable solution for this problem [187]. Other diagnostic panels are: ThyroidPrint^®^ (10-gene classifier) [188], ThyroSeq v3 (DNA- and RNA-based NGS assay analyzing 112 genes) [189], RosettaGX Reveal, and ThyraMIR/ThyGenX™, which are respectively 24-miRNA and 10-miRNA (with 8 DNA mutations) expression tests [190,191].

### 4.4. Intratumoral Heterogeneity (ITH) and Tumor Microenvironment (TME)-Related Research

Most of human cells have identical DNA material, despite the differences at the level of gene expression. For example, transcriptome and protein landscapes of the liver vary significantly from those of the heart. In addition, such cellular differences can be distinguished within the same organ, e.g., neurons and glial cells of the brain. These differences in gene expression within particular subpopulations of cells reflect their distinct morphologies, functions, regenerative capacities, etc. Thus, profiling of transcriptome of individual cells—single-cell RNA-Seq (scRNA-Seq)—provides a multidirectional insight into specific cell’s functions—also in cancer. The scRNA-Seq is valuable but challenging method preceded by dissociation of heterogenous tissue and optimized filtering [192]. To date, this approach has enabled identifications of novel cell populations and cell-cell interactions. The characterization of ITH reveals the molecular background for resistance to the cancer treatment, the dynamic of tumor growth and its potential of relapse [193]. Several works show the power of scRNA-Seq to distinguish the ITH. The study of Sharma et al. revealed the multi-level tumor evolution of lung squamous cell carcinoma based on cell subclones closely related to tumor-immune cell interactions [194]. In the study of Wu et al., the immunological interactions between breast cancer cells and TME were proved, showing the ability of scRNA-Seq for analyzing tumor in a dynamic state [195].

Intra-tumor heterogeneity generates serious complications, including resistance to therapy. In addition to the different subpopulations forming a tumor bulk, the response to the treatment and whole disease course are dependent, among others on the interactions within TME. It consists of complex ecosystem of stromal cells, such as cancer-associated fibroblasts (CAFs), immune cells, endothelial cells and others of lesser importance. As mentioned above, TME components actively influence tumor cell proliferation. For example, cytokines secreted by CAFs participate in signal pathways in cancer cells [196]. Moreover, the immune compartment of TME acts immunosuppressively, creating a barrier between cancer cells and the host’s immune system [197]. The investigation of mechanisms by which TME affects cancer biology is possible through leverage of gene expression profiling.

The technology which has been successfully applied in the studies of TME of the recent years is RNA-seq [198]. It has become an integral tool of immunogenomics and serves today for analysis of gene expression patterns associated with the presence of different populations of immunological cells in the cancerous tissue [199]. Identification of distinct immune and stromal cells subtypes enables not only for understanding of different metabolic phenotypes of tumors, but also for the assessment of immune receptor repertoire in purpose of precision cancer immunotherapy [200]. The T-cell receptor (TCR)/B-cell receptor (BCR) sequences on tumor cells provide an information about specificity of immune response in TME. This landscape of TCRs/BCRs may serve as a predictive biomarker of the effectiveness of immunotherapy [201,202]. However the validation cohorts of these gene signatures remains to be seen.

### 4.5. RNA-Based Therapeutics

Dynamic growth of knowledge on transcriptome has changed a simple understanding of RNA as an intermediary between DNA and protein to a whole variety of molecules with numerous functions and a potential of regulation of gene expression. Thus, particular RNAs can be used as therapeutic targets as well. The greatest progress in terms of RNA-based therapies has been made in regard to miRNA.

miRNAs, which selectively induce mRNA degradation and translation inhibition, present an abnormal expression within cancerous tissues. Depending on their functions, miRNAs may act as an oncogenes (also known as oncomiRs—Onc miRNAs) or tumor suppressors (TS miRNAs) [203]. OncomiRs (e.g., miR155 and miR21) promote cell transformation, carcinogenesis, and metastasis by blocking the expression of tumor suppressor genes, and hence can be used as therapeutic targets in cancer treatment [204,205,206]. In turn, TS miRNAs suppress the translation of mRNAs involved in oncogenic pathways. Many studies have confirmed that the expression of TS miRNAs are downregulated in cancer [207,208]. Therefore, miRNAs can be considered as an early indicator for diagnostic purposes and patient prognosis, as well as effective target for therapeutic strategies in oncology [203].

There are two main approaches for regulating miRNA activities: suppression of expression of oncogenic miRNAs, and restoration of the loss of TS miRNAs’ expressions [203,209]. Inhibition of oncomiRs’ expressions can be achieved through the use of antisense oligonucleotides with 2′-O-methyl groups [210,211,212], 2′-O-methoxyethyl groups [213] and other linkages blocking exonuclease degradation, such as *N*,*N*-diethyl-4-(4-nitronaphthalen-1-ylazo)-phenylamine [214]. Another strategy is an application of locked nucleic acid (LNA) anti-miRNA oligonucleotides with complementary sequences to target miRNAs [206,215]. Currently, two clinical trials, i.e., SOLAR (ClinicalTrials.gov identifier: NCT03713320; miRagen Therapeutics, Inc., Boulder, CO, USA) and PRISM (enrolling; ClinicalTrials.gov identifier: NCT03837457), investigate the efficacy and safety of Cobomarsen (MRG-106)—an LNA antimiR^®^ inhibitor of miR-155 in the treatment of cutaneous T-cell lymphoma (CTCL), adult T-cell lymphoma (ATLL), chronic lymphocytic leukemia (CLL) and diffuse large B-cell lymphoma (DLBCL) [216,217,218].

The approach focusing on restoration of down-regulated TS miRNAs is called miRNA replacement therapy [219]. It is based on delivery of key miRNAs for carcinogenesis using either viral or non-viral vectors [220]. Viral platforms have been found to be insufficient due to triggering of immune response [221]. Non-viral vectors include: inorganic miRNA platforms (e.g., Fe_3_O_4_-based nanoparticles [222], gold or nanodiamonds platforms [223], and silica-based systems [224]), polymer-based miRNA delivery systems (such as polyamidoamine dendrimers—PAMAMs [225,226], or atelocollagen [227,228]) and lipid-based platforms [229,230].

Considering the increase in RNA-focused studies over the recent years, the specific knowledge about RNA may result in introduction of clinical, RNA-based techniques [231]. Research efforts have focused on overcoming limitations related to RNA therapeutics: low bioavailability, poor cellular uptake or rapid elimination and excessive immune response [232,233]. Nevertheless, targeting RNAs networks in cancer present strong potential for future clinical utility. Therefore, a growth in RNA therapeutics in the next several years is highly expected.

## 5. Conclusions

Transcriptomics undoubtedly deserves to be considered as contemporary “game-changer” in oncology on both molecular and clinical levels. In recent years, the revolution in the understanding, research, and clinical implementation of RNA-based techniques can be observed. The constant development of molecular technologies used for transcriptome analysis and implementation of NGS platforms considerably changed the landscape of RNA world research. RNA-seq is continuously gaining larger role in the clinical care of cancer patients, as well as preclinical research and basic science. It enables an insight into the molecular mechanisms underlying cancer, which has given rise to numerous trials related to transcriptome-based personalized oncology. The increasing availability of transcriptome profiling enables the introduction of transcriptomics in clinics. To date, several prognostic and predictive gene expression assays, as well as cancer biomarkers, have been utilized in clinical oncology. The change of the paradigm of cancer classification—from histopathological and clinical to molecular, opens a more personalized perspective for tumor diagnostics and therapy. The further advancement of transcriptomeprofiling will allow for standardization and the cost reduction of its analysis, which will be the next step for transcriptomics to become a canon of contemporary cancer medicine.

## Figures and Tables

**Table 1:** Examples of transcriptome-based molecular classification of particular malignancies.

Cancer Type Based on Origin Tissue	Molecular Classification		Reference
**Breast cancer**	ER+/luminal-like		[87]
	Basal-like		
	Erb-B2+		
	Normal-like		
	Luminal A		[91]
	Luminal B		
**Colorectal cancer**	CMS1 (microsatellite instability immune)		[95]
	CMS2 (canonical)		
	CMS3 (metabolic)		
	CMS4 (mesenchymal)		
**Gastric adenocarcinoma**	EBV positive		[111]
	MSI high		
	GS		
	CIN		
**Glioblastoma**	prognostic subtypes	Poor (invasive)	[112]
		Favorable (mitotic)	
		Intermediate	
	Proneural		[113]
	Mesenchymal		
	Proliferative		

**Table 2:** Potential cancer biomarkers discovered through transcriptome analysis.

Cancer Type	Biomarkers	Expression in Cancer Tissue *	Reference
Breast cancer	miR-126-5p, miR-144-5p, miR-144-3p, miR-301a-3p, miR-126-3p, miR-101-3p, miR-664b-5p	up/down	[142]
	LINC00657 (lncRNA)	up	[143]
Colorectal cancer	ABCD3	down	[144]
	piR-5937, piR-28876, piR-23210, piR-32159	down	[140]
	miR-17-92a, miR-135	up	[145]
	miR-21	up	[141]
Esophageal cancer	CHI3L1, MMP13, SPP1	up	[128]
Gallbladder carcinoma	*BIRC5*	up	[146]
Gastric cancer	miR-106b, miR-20a, miR-21, miR-221, miR-451	up	[132]
	miR-17-5p, miR-21, miR-106a, miR-106b	up	[147]
	miR-1, miR-20a, miR-27a, miR-34a, miR-423-5p	up	[148]
	piR-651	down	[138]
Glioblastoma multiforme	Thymosin β4 (TMSB4X), S100A10	up	[149]
	miR-20a, miR-106a	up	[150]
Hepatocellular cancer	piR-013306	up	[136]
Hogdkin lymphoma	piR-651	down	[151]
Melanoma	Ro-aassociated Y RNA (YRNAs): RNY3P1, RNY4P1, RNY4P25 (upregulation) miR-320a-3p, miR-134-5p (downregulation)	up/down	[152]
	miR-21	up	[153]
Multiple myeloma	piR-823	up	[154]
Non-small cell lung carcinoma	4-miRNA	up	[131]
	snoRNA	up	[130]
	34 miRNA expression signature	up/down	[155]
Ovarian cancer	IGFBP-4	up	[117]
Pancreatic cancer	*KRAS* mRNA (as salivary biomarker)	up	[156]
	LAMC2	up	[157]
Renal cancer	PIWIL1, piR-823	down	[158]

* up—upregulated; down—downregulated.

## Data Availability

Not applicable.
